# Simultaneous Temperature and Relative Humidity Measurement Using Machine Learning in Rayleigh-Based Optical Frequency Domain Reflectometry

**DOI:** 10.3390/s24247913

**Published:** 2024-12-11

**Authors:** Mateusz Mądry, Bogusław Szczupak, Mateusz Śmigielski, Bartosz Matysiak

**Affiliations:** Faculty of Information and Communication Technology, Wroclaw University of Science and Technology, Wybrzeże Wyspiańskiego 27, 50-370 Wrocław, Poland; boguslaw.szczupak@pwr.edu.pl (B.S.); 260457@student.pwr.edu.pl (M.Ś.); 252757@student.pwr.edu.pl (B.M.)

**Keywords:** distributed fiber sensors, temperature sensor, humidity sensor, optical fiber sensors, machine learning

## Abstract

This paper presents, for the first time to the best of our knowledge, simultaneous temperature and relative humidity (RH) measurement using a machine learning (ML) model in Rayleigh-based Optical Frequency Domain Reflectometry (OFDR). The sensor unit consists of two segments: bare and polyimide-coated fibers, each with different sensitivities to temperature. The polyimide-coated fiber is RH-sensitive, unlike the bare fiber. We propose the ML approach to avoid manual post-processing data and maintain relatively high accuracy of the sensor. The root mean square error (RMSE) values for the 3 cm length of the sensor unit were 0.36 °C and 1.73% RH for temperature and RH, respectively. Furthermore, we investigated the impact of sensor unit lengths and number of data points on RMSE values. This approach eliminates the need for manual data processing, reduces analysis time, and enables accurate, simultaneous measurement of temperature and RH in Rayleigh-based OFDR.

## 1. Introduction

Distributed optical fiber sensors (DOFSs) are of great interest due to the possibility of detecting changes in temperature and strain along their entire length [1,2]. Because of the unique properties of light transmission in optical fibers, they can operate in harsh environments. These sensors are widely used in various branches of industry, including structural health monitoring, environmental parameter detection or pipeline surveillance [3]. Among these, Rayleigh-based distributed fiber sensors stand out for their high spatial resolution and short measurement time, especially in optical frequency domain reflectometry (OFDR) [4]. One of the most important environmental parameters is relative humidity (RH) used for different purposes, i.e., structural health monitoring (SHM), electronics, food processing and storage applications, pharmaceutical manufacturing, or agricultural production [5]. Therefore, Thomas et al. presented an RH sensor based on the OFDR setup with cm-scale resolution and a sensitivity of 1.3 µε/% RH [6]. This sensitivity could be improved by applying a thicker layer of polyimide (PI), which was experimentally proven for a thickness of 876 µm [7]. The maximal sensitivity was then 38.5 µε/% RH. However, the PI-coated fibers are sensitive not only to RH but also to temperature. OFDR setups with conventional fibers cannot distinguish between temperature and RH influences based only on the spectral shift in scattering light. This cross-sensitivity issue is crucial because temperature and RH are common environmental parameters that could change simultaneously. To address the measurement of temperature and RH, some methods have been proposed [8,9,10]. One approach involves a multi-fiber tandem connection with varying thicknesses of PI for simultaneous temperature and RH measurement [8]. Based on a comprehensive analysis, two PI-coated fibers were selected and connected to create the sensor unit. Using a matrix of sensitivities to the temperature and RH of these two fibers, simultaneous measurement could be possible. In another study, a PI-coated polarization-maintaining fiber (PMF) was used as a sensor for discrimination between temperature and RH [9]. However, this method is time-consuming, requires a large data analysis, and limits the achievable gauge length to 5 cm. Another approach enables the simultaneous sensing of RH and temperature by two parallel fibers’ configuration [10]. The use of PI-coated and copper-coated fibers in a parallel configuration allows for the discrimination of temperature and RH by the analysis of spectral shifts for two lines. However, each of the mentioned works relies on the manual analysis of the measurement data, which could be time-consuming and lead to undesired errors.

In recent years, machine learning (ML) has been willingly used in optical fiber technology. ML models in integration with FBGs [11,12], Vernier setups based on Sagnac loops [13], Fabry–Perot interferometers [14,15,16], or BOFDA [17,18,19,20] have been proposed in the literature. All of them used different ML approaches to enhance measurement data time processing and accuracy and avoid manual calculations. In the OFDR-based sensor setup, the ML model was used to improve strain resolution [21]. The use of a deep learning approach was also presented for the measurement of temperature and strain in single-mode fiber (SMF) by ϕ-PA-OFDR [22].

However, none of the presented papers demonstrate the possibility of measuring temperature and RH based on OFDR using an ML approach. Thus, in this paper, for the first time, to the best of our knowledge, a simultaneous temperature and RH distributed fiber sensor based on OFDR and the ML approach is proposed. It combines the efficiency of the OFDR setup and the advantages of ML for prediction based on measurement data. The well-established and fast ML linear regression model was used. The sensor principle operation is based on a PI-coated fiber in series with a bare one. By utilizing a single-mode fiber optic sensor that combines uncoated and polyimide-coated sections, we can exploit the distinct physical interactions these sections have with temperature and humidity to separate their effects on the spectral shift. This approach simply uses different sensitivities of the fibers to temperature and RH. The PI-coated fiber is sensitive to temperature and RH; otherwise, the bare fiber is sensitive only to temperature. This allows for simultaneous measurement based on OFDR with the ML model, which enhances data processing and prevents improper manual data analysis. Root mean square errors (RMSEs) are analyzed, as well as training time consumption, regarding different sensor lengths and numbers of data points.

## 2. Materials and Methods

The optical backscatter reflectometer (OBR) employs swept-wavelength interferometry (SWI) to measure Rayleigh backscatter along the length of an optical fiber with high spatial resolution [23]. Rayleigh backscatter arises from random fluctuations in the refractive index profile along the fiber, which can be modeled as a continuous, weak fiber Bragg grating (FBG) with a random period. Both the physical length and the refractive index of the fiber are inherently sensitive to environmental parameters such as temperature and strain. This allows the OBR to detect and measure these changes through a cross-correlation operation between the reference and measured spectra. The spectral response to changes in strain (Δ*ε*) or temperature (Δ*T*) is similar to that of a conventional FBG, characterized by a shift in wavelength (Δ*λ*) or frequency (Δ*ν*). Thus, it could be expressed by the following equation [24]:

(1) $$\frac{\Delta \lambda }{\lambda }=-\frac{\Delta v}{v}=K_{T}\Delta T+K_{\varepsilon }\Delta \varepsilon$$

where *λ* and *ν* refer to optical wavelength and frequency, and *K_T_* and *K_ε_* represent the sensitivities to temperature and strain, respectively. Similarly to PI-coated FBGs, polyimide-coated fibers could also be used for RH sensing [25]. An increase in RH causes the polyimide to absorb moisture, leading to its expansion, which results in a strain on the fiber. The dependence of the thickness on sensitivity is observed. When the PI coating is thicker, the fiber is more sensitive to RH. Bare fiber, without an additional coating, is insensitive to RH. Assuming that Rayleigh scattering could be modeled as a continuous, weak FBG, the equation for a spectral shift due to humidity changes could be expressed as follows [25]:

(2) $$\frac{\Delta \lambda }{\lambda }=(1-P_{e})\varepsilon +[(1-P_{e})\alpha +\xi ]\Delta T$$

where *λ* refers to wavelength, *P_e_* corresponds to the photoelastic constant of the optical fiber, *ε* is the strain induced on the fiber due to humidity, *α* refers to the thermal expansion coefficient of the coated fiber, *ξ* is the thermo-optic coefficient of the fiber, and *ΔT* is the temperature change. However, the standard approach requires manual extraction and analysis of data for each segment to establish the temperature and RH relationship. In contrast, our method removes the need for such calculations and matrix formulations. Instead, we developed an ML model that directly processes spectral shifts to provide information on temperature and RH. This approach uses raw data, which are then filtered and processed by our ML model. The relationships between different states of temperature, RH, and spectral shifts are fully captured by the ML model, enabling direct and efficient processing of raw data.

### 2.1. Experimental Setup

**Figure 1:**
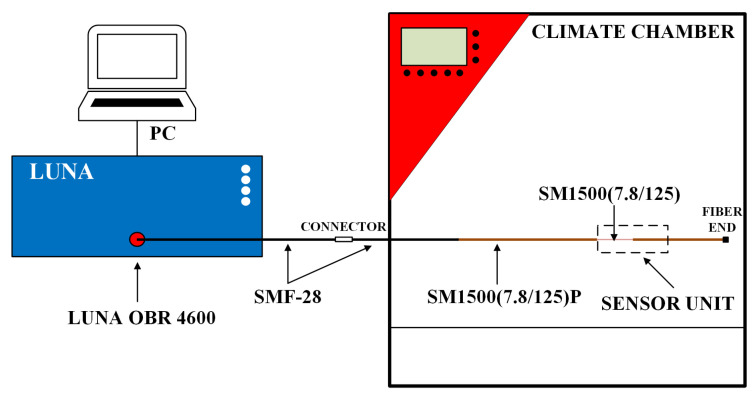
The scheme of the experimental setup consisted of LUNA OBR 4600 as Rayleigh-based OFDR, a PC (Personal Computer) with dedicated software, SMF-28, bare fiber—SM1500(7.8/125), PI-coated fiber—SM1500(7.8/125P), and the climate chamber. The sensor unit consists of bare and PI-coated fibers.

The experimental setup consists of LUNA OBR 4600 (Luna, Roanoke, VA, USA) as the Rayleigh-based OFDR connected to the computer with dedicated software for measurement, optical fibers, and climate chambers, which is shown in Figure 1.

The sensor unit contains two segments: bare and PI-coated fibers in series. The parameters of commercially available PI-coated SMF (SM1500(7.8/125)P, Fibercore, Southampton, UK) given by the manufacturer are listed in Table 1. Meanwhile, the bare fiber was simply prepared by stripping off the polyimide buffer from the mentioned fiber.

**Figure 2:**
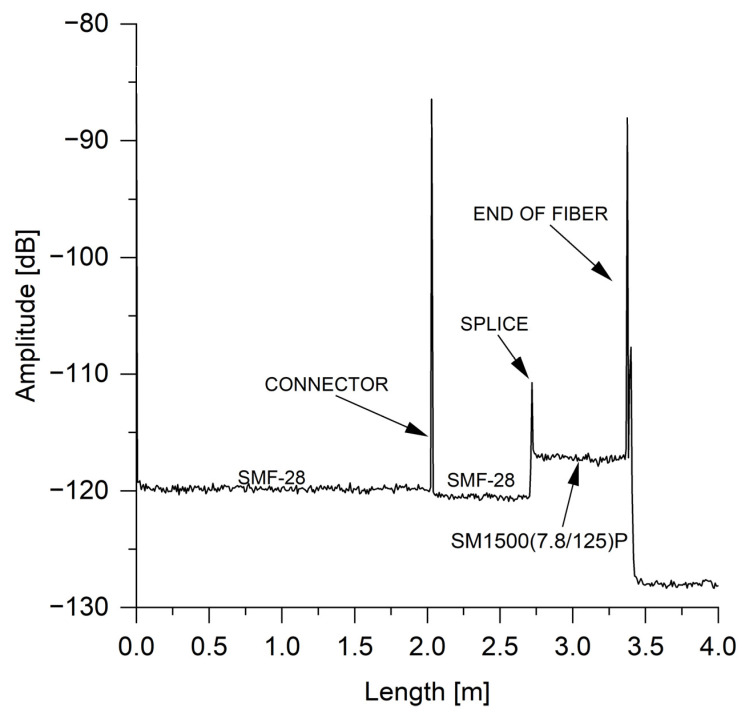
The backscattered trace from LUNA OBR 4600 to the end of the fiber line.

The sensor unit was prepared without any splices between the fiber segments. Part of the fiber line was placed inside the climate chamber (KMF 115, BINDER GmbH, Tuttlingen, Germany), which had temperature and RH fluctuations in the range of 0.1 to 0.3 °C and within 2.5%, respectively. Climate chamber KMF 115 is a well-established device designed to maintain highly stable test conditions across the entire testing area, as specified by the manufacturer. The climate chamber is equipped with built-in temperature and humidity sensors, as well as an advanced control system (APT.line™ controller), which ensures the uniform distribution of temperature and humidity within the chamber. Additionally, other climate chambers have also been employed in similar studies [7,10], which further confirms the reliability of this approach for controlled environmental testing. Fibers could be protected against undesired external forces by an outer tube, for instance, by a spring hose, meanwhile allowing them to interact with the environment [8]. During this study, measurements were made for RH in a range of 20–70% RH with steps of 10% RH and a temperature range of 30–80 °C with steps of 10 °C. Based on the combined findings from studies [26,27], the measurement range for RH can be extended to 10–97% RH, and the temperature range can be increased to 350 °C, as specified by the manufacturer of the fibers. Furthermore, our ML approach is also applicable to this extended measurement range, as it relies on the distribution of spectral shifts along the fiber’s length. The reference state was 20% RH and 30 °C. For each state of temperature and RH, the measurements were repeated thirty times to prepare the input data set for ML training. Additionally, for each state of the RH level, the stabilization time before measurement was one hour. The LUNA OBR 4600 was set for a full laser wavelength scanning range (1545.445–1588.034 nm). An example of reflection trace, which shows the optical fiber link at a temperature of 30 °C and 40% RH, is shown in Figure 2.

After averaging, the average backscatter level for SMF-28 after the connector was measured at −120.52 dB, while for SM1500 (7.8/125), it was −117.21 dB. Consequently, the gain was approximately 3.31 dB. This increase is attributed to the higher concentration of germanium in the SM1500 (7.8/125) fiber core, resulting in a higher numerical aperture, as thoroughly investigated in one of our studies [28]. Therefore, the Rayleigh scattering level was higher for the SM1500 (7.8/125) fiber than for the SMF-28 fiber. High-scattering fibers, as well as nanoparticle-doped fibers, can be employed in OFDR setups for sensing purposes. These fibers enhance the Rayleigh scattering signal, allowing for the differentiation of multiple fiber lines using the Scatter Level Multiplexing (SLMux) scheme. Two primary approaches are used to enhance Rayleigh scattering: the post-fabrication treatment of fibers and fiber design with various dopants [29,30]. Post-fabrication treatments, such as UV exposure [31,32], can increase the amplitude of Rayleigh scattering in optical fibers. Other methods use fibers with various dopants, which could also boost the Rayleigh scattering, for instance, magnesium oxide (MgO) [33,34], zirconia [35], or calcium [36].

### 2.2. Machine Learning Approach

**Figure 3:**
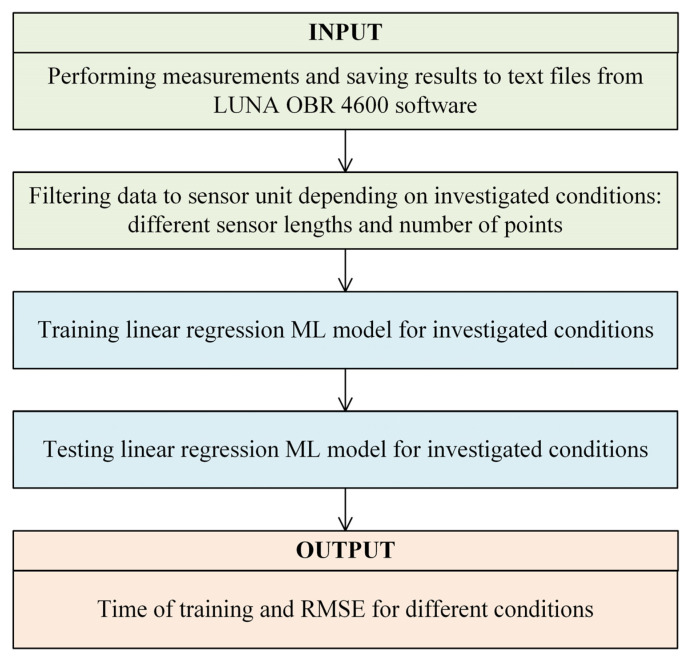
The algorithm for measurement data processing in this study.

The flow of data processing using ML is presented in Figure 3.

The software code was written in *Python* (ver. 3.11.4) and exploited a well-known library, *scikit-learn*. First, the data were loaded and initially filtered to include only the sensor unit. The sensor unit was trained using a linear regression ML model. This model was chosen due to the linear relationship between spectral shifts and changes in temperature or RH, as shown in the literature [25,26,28]. Linear regression offers a straightforward and computationally efficient approach to modeling this relationship. While more complex models like neural networks were considered, preliminary research indicated that the linear regression model provided sufficient accuracy with minimal computational overhead. The selected linear regression model utilizes the least squares method to minimize the sum of squared differences between observed and predicted values. In this study, the measured parameters were target output variables of linear regression. A total of 35 states (temperature and RH) without reference (20% RH and 30 °C) were investigated, with 30 repeated measurements made for each state. This resulted in 1050 raw data files. A matrix of spectral shifts and length positions was created as input data. The tests were performed for different gauge lengths of sensor units and different numbers of data points. The software code was prepared for experiments to filter sensor units to smaller sizes (gauge length) and select every examined measurement point from raw data (number of data points). Depending on the number of measurement points and the gauge length, different datasets were used for training and testing. Leave-one-out cross-validation was implemented for testing purposes, which provided an accurate estimation of model performance as the model did not encounter testing states during the training phase. The measured outputs included training time and RMSE values—a widely used metric for quantifying the difference between values predicted by a model and the actual observed values in regression tasks. The training time refers to the entire procedure regarding the training and testing model.

## 3. Results and Discussion

**Figure 4:**
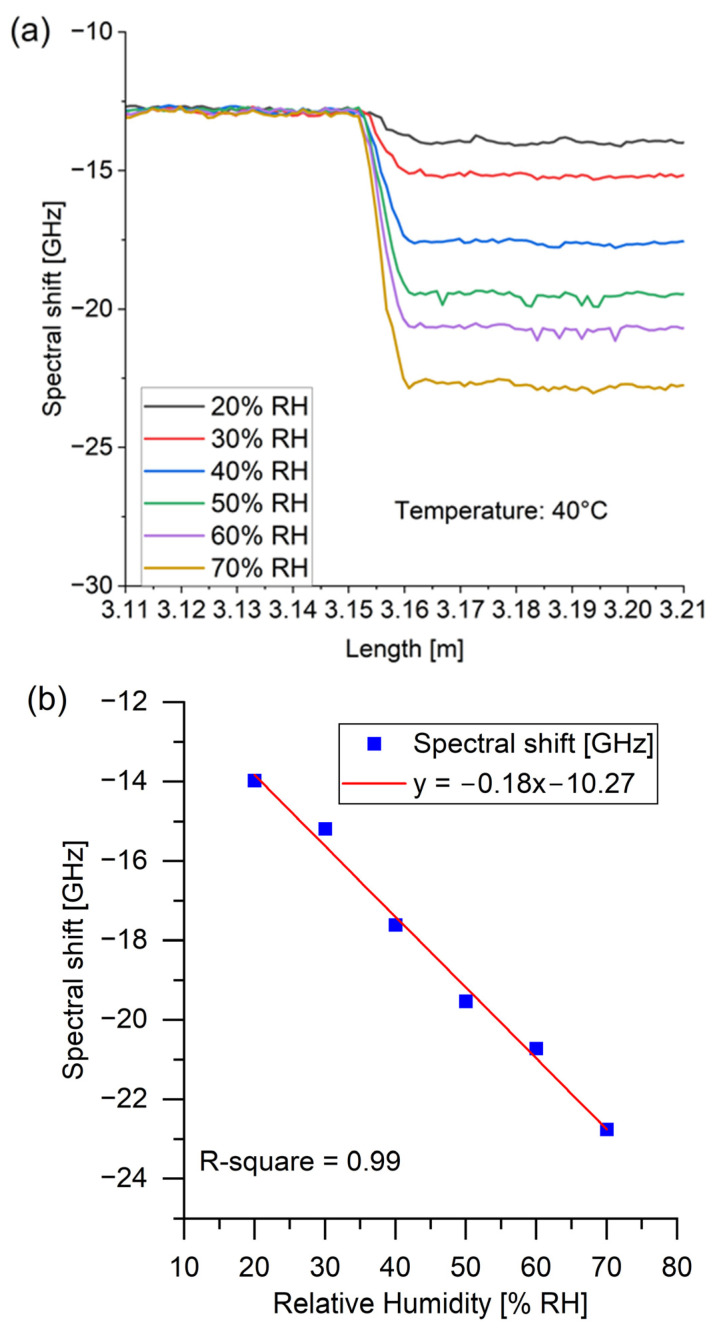
(**a**) The exemplary spectral shift as a function of fiber length (limited to the investigated section) for different RH values at a constant temperature of 40 °C. (**b**) Spectral shift values as a function of RH changes.

Measurements of spectral shifts have been performed for different applied temperature and RH values. The reference measurement was carried out for a temperature of 30 °C and 20% RH. The exemplary spectral shifts for different RH states (20–70% RH) and constant temperature (40 °C) are presented in Figure 4 and show that a spectrum shift was observed for the PI-coated fiber segment of the sensor unit. The approximated RH sensitivity of −0.18 GHz/% RH refers to PI-coated fiber, while, in the meantime, the bare fiber did not respond to RH changes. This behavior is consistent with our previous research [28]. The linear dependency between spectral shift values and RH changes is also shown in Figure 4, with an R-squared value of 0.99, which is consistent with the literature [26,27].

**Figure 5:**
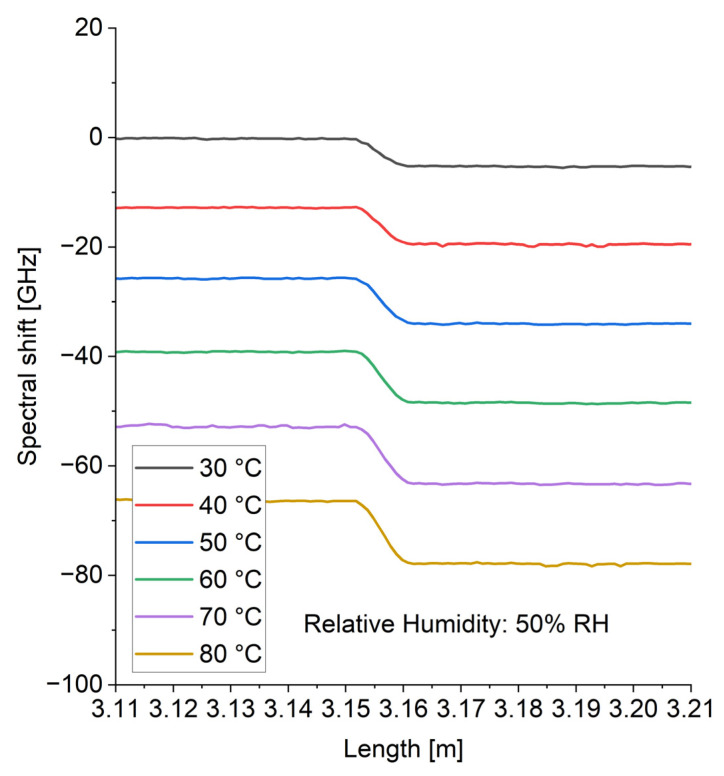
The exemplary spectral shift as a function of fiber length (limited to the investigated section) for different temperature values at constant 50% RH.

Similarly to the changes in RH, the exemplary spectral shifts for different temperatures (30–80 °C) and constant RH (50% RH) are presented in Figure 5. The spectral shift was observed for temperature changes. A slight shift was observed for the PI-coated fiber compared to the bare fiber. This resulted from the polyimide coating, which underwent thermal expansion and induced strain, resulting in a higher spectral shift compared to the bare fiber [28]. Based on the measurement data presented in Figure 5, the temperature sensitivities were −1.33 GHz/°C and −1.45 GHz/°C for bare fiber and PI-coated fiber, respectively.

**Figure 6:**
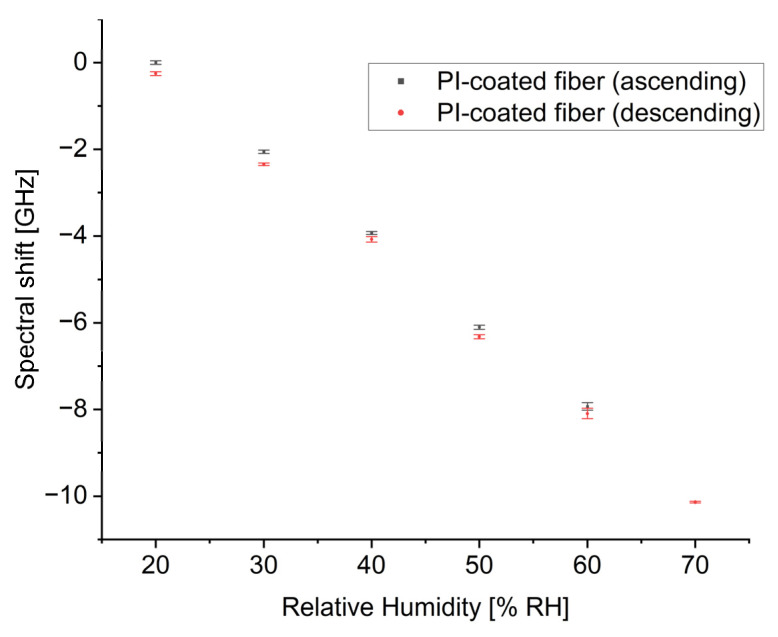
The spectral shift as a function of ascending and descending RH for PI-coated fiber.

The hysteresis effect was also investigated with respect to RH in the climate chamber. The responses of the PI-coated fiber were examined during both ascending and descending RH levels within a range of 20–70% RH at a constant temperature of 30 °C. The averaged spectral shifts based on repeated measurements, along with their standard deviations, indicated as error bars, in a function of increasing and decreasing RH values, are presented in Figure 6.

**Figure 7:**
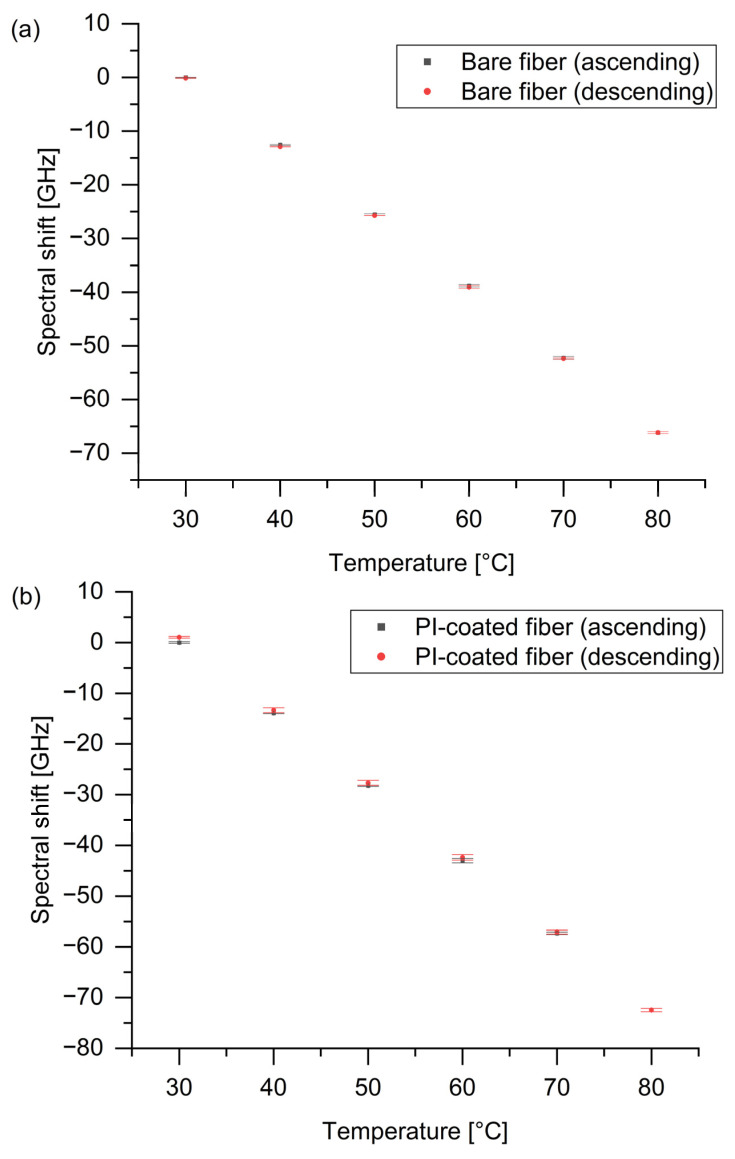
The spectral shift for ascending and descending temperatures in a range of 30–80 °C for (**a**) bare fiber and (**b**) PI-coated fiber.

Based on the repeated measurements and corresponding sensitivity, the maximum averaged observed difference for PI-coated fiber was 0.29 GHz, which relates to approximately 1.61% RH. A similar experiment was performed for temperature measurements. The bare and PI-coated fiber responses were examined for ascending and descending temperatures in a range of 30–80 °C at a constant 40% RH based on repeated measurements. Averaged results of spectral shifts and related temperatures along with their standard deviations, indicated as error bars, are presented in Figure 7. The maximum difference between increasing and decreasing values was 0.33 GHz for the bare fiber and 1.04 GHz for the PI-coated fiber, corresponding to 0.25 °C and 0.72 °C, respectively.

**Figure 8:**
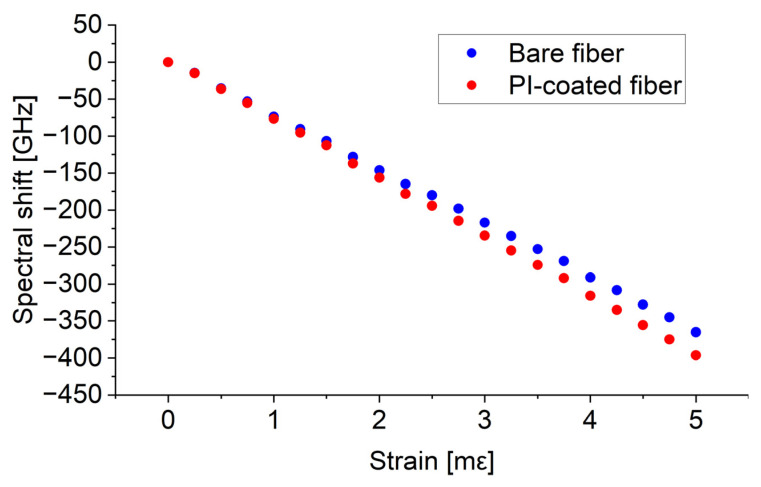
The spectral shift as a function of strain for bare and PI-coated fibers.

Additional measurements were also conducted to assess the cross-sensitivity to strain. The PI-coated fiber, along with the bare fiber, was mounted on two dual-axis micropositioner stages, each equipped with a micrometer screw with a resolution of 10 µm. The temperature was maintained at the level of 21 °C. During the experiment, the fiber was stretched, and the results were recorded. As expected, the sensor exhibited strain sensitivity. The bare fiber had strain sensitivity of −72.87 GHz/mε, while the PI-coated fiber showed slightly higher sensitivity of −79.65 GHz/mε. The strain responses for both types of fibers are presented in Figure 8. The sensitivities to strain were greater than those to temperature and RH. Strain sensitivity is a common issue in many optical fiber sensors, including those designed to measure other parameters. These sensors often exhibit sensitivity to both temperature and strain. In this study, we emphasized the importance of considering strain during the implementation stage. For effective operation, the fiber should interact with the external parameters of interest while being protected by an outer tube to prevent longitudinal strain. Therefore, in practical applications, adequate protection is necessary, as already highlighted in previous studies [8,37]. One approach involves shielding the sensor with appropriate packaging, such as a metal spring hose [8]. In another study, Alwis et al. proposed PEEK packaging for an optical fiber humidity sensor [37].

**Figure 9:**
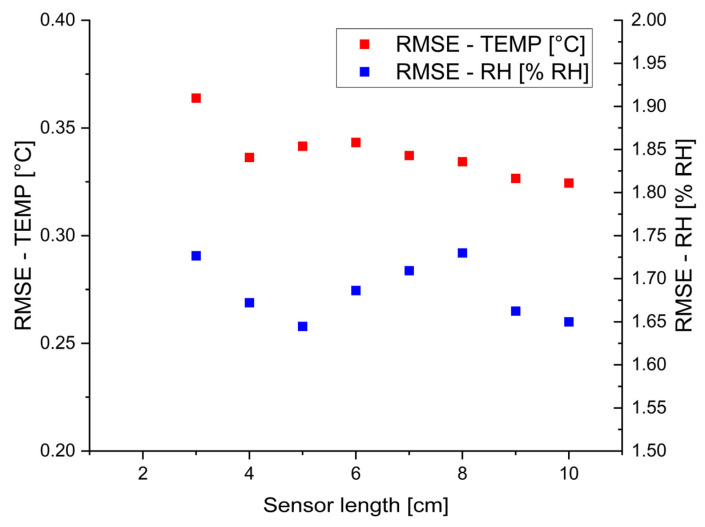
The RMSE values for different sensor lengths (3–10 cm) for temperature and RH, respectively.

Based on measurement data as input for the model, the RMSE values and the training time were calculated. Figure 9 shows the relationship between sensor lengths and RMSE values for temperature and RH.

The RMSE values for temperature were almost unchanged (from 0.32 to 0.36 °C) when the sensor length decreased from 10 cm to 3 cm. A similar trend occurred for RH with an RMSE value of 1.73% RH for a 3 cm sensor unit length. The overall changes across the investigated sensor unit lengths were minimal (0.09% RH). These results suggest that RMSE for RH was also almost independent of the examined range of sensor unit lengths. Therefore, applying the shortest (3 cm) sensor length did not worsen the performance of the proposed model. Instead, the investigation of the 2 cm sensor length led to higher error values (RMSE values: 1.22 °C and 9.45% RH); thus, a 3 cm sensor length was used as a boundary condition in the experiments. The minimization of RH errors could be further improved, probably by using other coatings or types of fibers with a higher susceptibility to RH changes. For example, the use of a thicker PI coating could improve RH sensitivity [7,25]. One of the most important parameters is also the time of data processing. The ML training and testing phases were executed on a PC equipped with an AMD Ryzen 7 5800H processor and 16 GB of RAM. Experiments revealed that an increase in sensor length implies longer time processing of the proposed model (from 4 ms for a 3 cm sensor length up to 34 ms for a 10 cm length). This is straightforwardly related to more data points that need to be exploited in the training stage. However, it clearly shows that the training model does now have a time-consuming nature. It is undoubtedly a much faster solution than manual data analysis.

**Figure 10:**
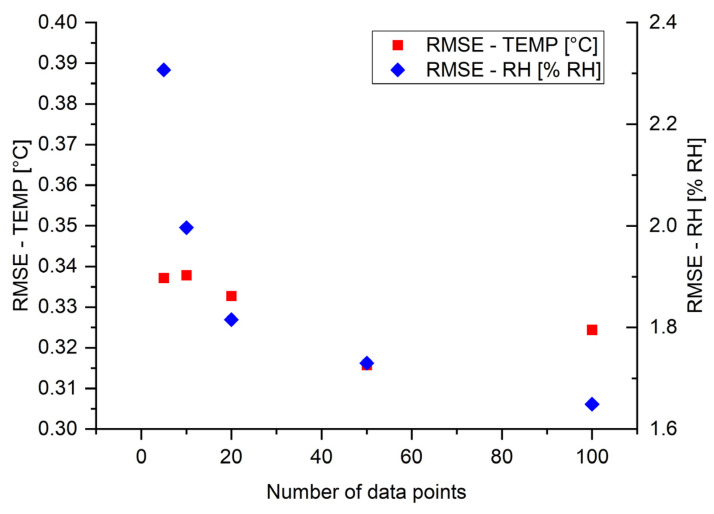
The RMSE values for different numbers of measurement points with constant value of sensor length (10 cm) for temperature and RH, respectively.

The additional investigation covered different numbers of points taken for training and testing models. Each measurement point was selected based on the experimental type (e.g., 1 in 2 indicates 1 point for every 2 points). Consequently, five different cases were investigated: 100 points (reference), 50 points (1 in 2), 20 points (1 in 5), 10 points (1 in 10), and 5 points (1 in 20). Experiments were carried out for a constant value of the input sensor length (10 cm). The RMSE values for temperature and RH as a function of the number of data points used in model training are presented in Figure 10. The analysis clearly demonstrates that the RMSE values for temperature remained relatively constant, ranging from 0.32 to 0.34 °C. Thus, it can be concluded that it is independent of the investigated number of data points. However, the RMSE values for RH changed significantly over the tested range from approximately 1.65% RH for 100 data points (reference) to 2.31% RH for 5 data points (1 in 20). RMSE values changed more rapidly with decreasing amounts of data. Nevertheless, it shows that both the gauge length and the number of data points could be reduced. Retrieving fewer measurement data points positively affects the processing time and the size of raw data storage files. However, it negatively affects prediction errors.

**Figure 11:**
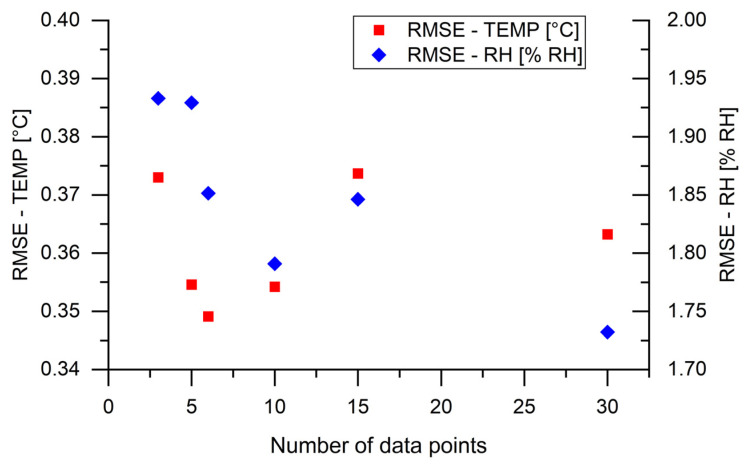
The RMSE values for different numbers of measurement points with constant values of sensor length (3 cm) for temperature and RH, respectively.

We also conducted experiments using a constant 3 cm sensor length and varying numbers of data points. Specifically, we investigated the following configurations: 30 points (1 in 1), 15 points (1 in 2), 10 points (1 in 3), 6 points (1 in 5), 5 points (1 in 6), and 3 points (1 in 10). The RMSE values for temperature and RH as a function of these data point ranges for the 3 cm sensor length are presented in Figure 11.

Based on the results, the RMSE values for temperature fluctuated between approximately 0.35 °C and 0.38 °C, while the RMSE for RH changed from 1.73% for 30 data points (reference) to 1.93% for 3 data points (1 in 10). These results highlight the huge advantages of the proposed ML approach. By using ML processing, the number of data points required can be significantly reduced, even for shorter sensor lengths, which leads to faster measurements and less data processing. This aligns with previous studies on ML approaches in fiber sensors [14,15,16]. Our investigation shows that the gauge length can be reduced to 3 cm, and even as few as three data points can be used for a single sensor unit. Additionally, the number of sensor units on a single fiber can be increased, and the ML approach can be applied to other sensor units with fewer data points.

The comparison of the proposed approach to other studies regarding the measurement of temperature and RH based on OFDR was also presented in Table 2.

This is the first time that the ML approach has been used in the OFDR setup for temperature and RH discrimination. The unique advantage over other studies is that this approach does not require manual calculations based on raw measurement data. The gauge length and prediction errors are comparable to those in other studies. The time to process the measurement data was very fast, even 4 ms for a sensor unit 3 cm in length. This factor is difficult to assess for other proposed setups as they were analyzed manually. Nevertheless, the proposed method is probably the most time-effective solution. In this study, only one fiber is required, without the need for additional optical couplers or switches, unlike in [10]. The presented approach involves the analysis of only spectral shift changes and the ML model for temperature and RH discrimination. An additional benefit of the proposed ML approach is its ability to reduce the amount of measurement data required for processing and predicting measurable parameters. Our results demonstrate that prediction errors do not change significantly even when fewer data points are used. The ML approach maintains accuracy and performs effectively with only a minimal number of data points, without significant loss in prediction quality. It eliminates the need for the manual analysis of spectra, which is required in classical methods. In traditional approaches, it is necessary to manually select and calculate sensitivity values before conducting further analyses, as discussed in the literature [38,39,40,41]. This process allows for the creation of matrix equations, which are then used to determine the desired parameters. In contrast, our approach only requires measurement data (spectral shifts as a function of length) from the OFDR system, without the need for manual analysis, such as sensitivity calculations for selected ranges. As a result, sensitivity matrix equations, commonly used in multi-parameter fiber sensing, are not required. This paper stands out among others in the literature due to its data processing techniques, accuracy, and sensor length.

## 4. Conclusions

Simultaneous temperature and RH sensing by an OFDR setup based on the ML model has been presented for the first time. It shows the possibility of discriminating between the two environmental parameters within one fiber line using an ML model. The analysis examined the effects of sensor lengths and the number of data points on RMSE values. The achieved RMSE values for the 3 cm sensor unit were 0.36 °C and 1.73% RH for temperature and RH, respectively. The proposed approach is beneficial for enhancing the post-processing measurement data and avoiding manual analysis. In the future, we plan to explore different sensor configurations and fiber types to simultaneously measure various parameters using machine learning models.

## Figures and Tables

**Table 1:** The selected fiber given by the manufacturer.

Type of Fiber	SM1500(7.8/125)P
Core/cladding diameter [μm]	7.8/125
Operating wavelength [nm]	1520–1650
NA @1550 nm (catalog card)	0.17
Type of coating	Polyimide
Coating thickness [μm]	15

**Table 2:** The comparison of proposed solution to other presented in the literature.

Ref	Configuration	Gauge Length	Machine Learning	Time of Data Processing	Prediction Errors
[8]	One fiber line (tandem)	5 cm	No	Not given	±5% RH, ±1 °C
[9]	One fiber line (PM fiber)	5 cm	No	Not given	2.75% RH, 0.54 °C
[10]	Two parallel fibers	1 cm	No	Not given	1.8% RH, 0.48 °C
This work	One fiber line (tandem)	3 cm	Yes	4 ms	1.73% RH, 0.36 °C

## Data Availability

The original contributions presented in this study are included in the article. Further inquiries can be directed to the corresponding author(s).
